# Purpura Fulminans in a Patient With Septic Shock due to Escherichia coli Bacteremia With Emphysematous Pyelitis

**DOI:** 10.7759/cureus.13249

**Published:** 2021-02-09

**Authors:** Maria Del Mar Morales Hernandez, Michael Carranza, Bijal Patel, Joshua Calvert, Ghania Masri

**Affiliations:** 1 Internal Medicine, University of Florida College of Medicine, Gainesville, USA; 2 Internal Medicine, University of Florida College of Medicine – Jacksonville, Jacksonville, USA

**Keywords:** purpura fulminans, disseminated intravascular coagulation, symmetric peripheral gangrene, escherichia coli

## Abstract

Purpura fulminans (PF) is a rapidly fatal disorder predominantly encountered in patients with an acquired deficiency of physiologic anticoagulants due to severe sepsis and septic shock with disseminated intravascular coagulation (DIC). This consumptive process eventually leads to widespread thrombosis, hemorrhagic necrosis, and gangrene. Rapid identification followed by aggressive management of the underlying etiology with a multidisciplinary team is critical to prevent long-term organ dysfunction, disability from amputation, and death. While bleeding is a common finding in DIC, anticoagulation must be considered if PF is present. We report a case of *Escherichia coli­*-associated emphysematous pyelitis leading to bacteremia, septic shock, and PF with small- and medium-sized vessel thrombosis and acral ischemia.

## Introduction

Purpura fulminans (PF) and symmetric peripheral gangrene (SPG) are rare manifestations of a severe underlying systemic coagulopathy characterized by rapidly evolving microthrombosis of dermal vessels and hemorrhagic epidermal necrosis. While PF and SPG are often used interchangeably, the lesions are distinguished by their non-acral or acral distribution, respectively [1,2]. Lesions often start out as erythematous macules, sometimes in a retiform pattern, and progress rapidly to hemorrhagic necrosis that may form bullae and are separated from uninvolved skin by an erythematous border [3,4]. Several clinical scenarios have been associated with the development of PF and SPG [4,5], the most common of which is acquired deficiency of physiologic anticoagulants in severe systemic bacterial infections associated with severe hepatic dysfunction and disseminated intravascular coagulation (DIC). PF may also be seen in neonates and young children with inherited PC or protein S (PS) deficiency, as an autoimmune post-viral syndrome, or following initiation of warfarin. The most frequently encountered bacterial cause of PF and SPG in adults is Pneumococcus [6]. Cases attributed to other pathogens such as *Escherichia coli* are rare, with only a handful of case reports and small case series described in the literature [7,8].

We present a case of a patient with features of PF and SPG caused by a severe *E. coli* infection leading to septic shock and DIC. This represents the first reported case of PF and SPG caused by *E. coli* emphysematous pyelitis; we highlight the differential diagnosis of purpura in septic patients and the role of anticoagulation in the medical management of DIC with systemic microvascular thrombosis.

## Case presentation

**Figure 1:**
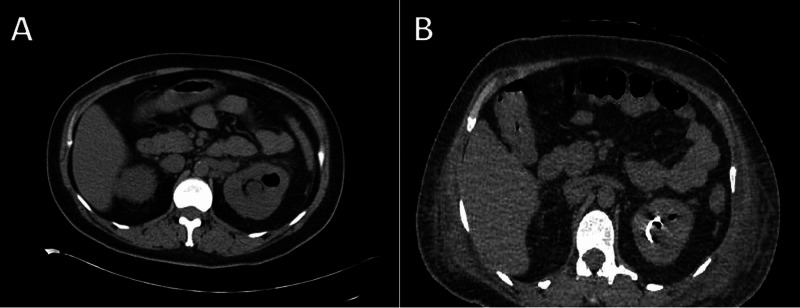
CT of the abdomen and pelvis demonstrating perinephric stranding and extensive pneumoureter with gas extending into the renal collecting system (A), which improved on repeat CT following emergent left ureteral stenting (B).

A 42-year-old female from Mexico presented to the emergency department for a five-day history of left flank pain associated with fever and vomiting. On examination, the blood pressure was 89/67, the heart rate 120 beats per minute, respiratory rate 18 breaths per minute, temperature 38 °C, and oxygen saturation 98% while breathing ambient air. Physical examination revealed left costovertebral angle tenderness, diminished radial and dorsalis pedis pulses bilaterally, and purple discoloration of the first, second, and fifth digits of her left hand and the first and fourth digits of the right hand. Her past medical history was significant only for hypertension, for which she took lisinopril. There was no personal or family history of clotting or bleeding disorders. Computerized tomography (CT) of the abdomen and pelvis revealed left emphysematous pyelitis (Figure 1A). Initial laboratory investigations revealed high anion gap metabolic acidosis with serum bicarbonate levels of 12 mmol/L (ref: 21-29 mmol/L), anion gap 31 mmol/L (ref: 4-16 mmol/L), hyperglycemia with glucose 446 mg/dL (ref: 71-99 mg/dL), beta hydroxybutyrate 6.1 mmol/L (ref: 0.2-2.8 mmol/L), hemoglobin 9.6 g/dL (ref: 12.0-16.0 g/dL), platelet count 70,000 (ref: 140,000-440,000/mm^3^), prothrombin time 22.4 seconds (ref: 11.9-14.3 seconds), international normalized ratio 2.0 (ref: 0.9-1.1), normal partial thromboplastin time, acute ischemic hepatitis with aspartate aminotransferase (AST) 64-1,052 IU/L (ref: 14-33 IU/L) and alanine aminotransferase (ALT) 32-550 IU/L (ref: 10-42 IU/L), with a predominantly direct hyperbilirubinemia 6.7 mg/dL(ref: 0.0-0.2 mg/dL), lactate dehydrogenase 429 IU/L (ref: 126-266 IU/L), a mildly elevated haptoglobin 202 mg/dL (ref: 30-200 md/dL), fibrinogen 615 mg/dL (ref: 186-461 mg/dL), and a peripheral smear without schistocytes. The patient was subsequently admitted to the intensive care unit for septic shock due to *E. coli* bacteremia, multisystem dysfunction marked by ischemic hepatitis, coagulopathy, and non-oliguric acute kidney injury, as well as new-onset diabetes mellitus with diabetic ketoacidosis. Broad-spectrum systemic antibiotics, intravenous insulin, and aggressive fluid resuscitation were initiated, followed by emergent ureteroscopy. Purulent material was visualized upon the instrumentation of the left ureter. She required 48 hours of norepinephrine administered via a central venous catheter, with invasive hemodynamic monitoring facilitated by a right radial artery catheter.

**Figure 2:**
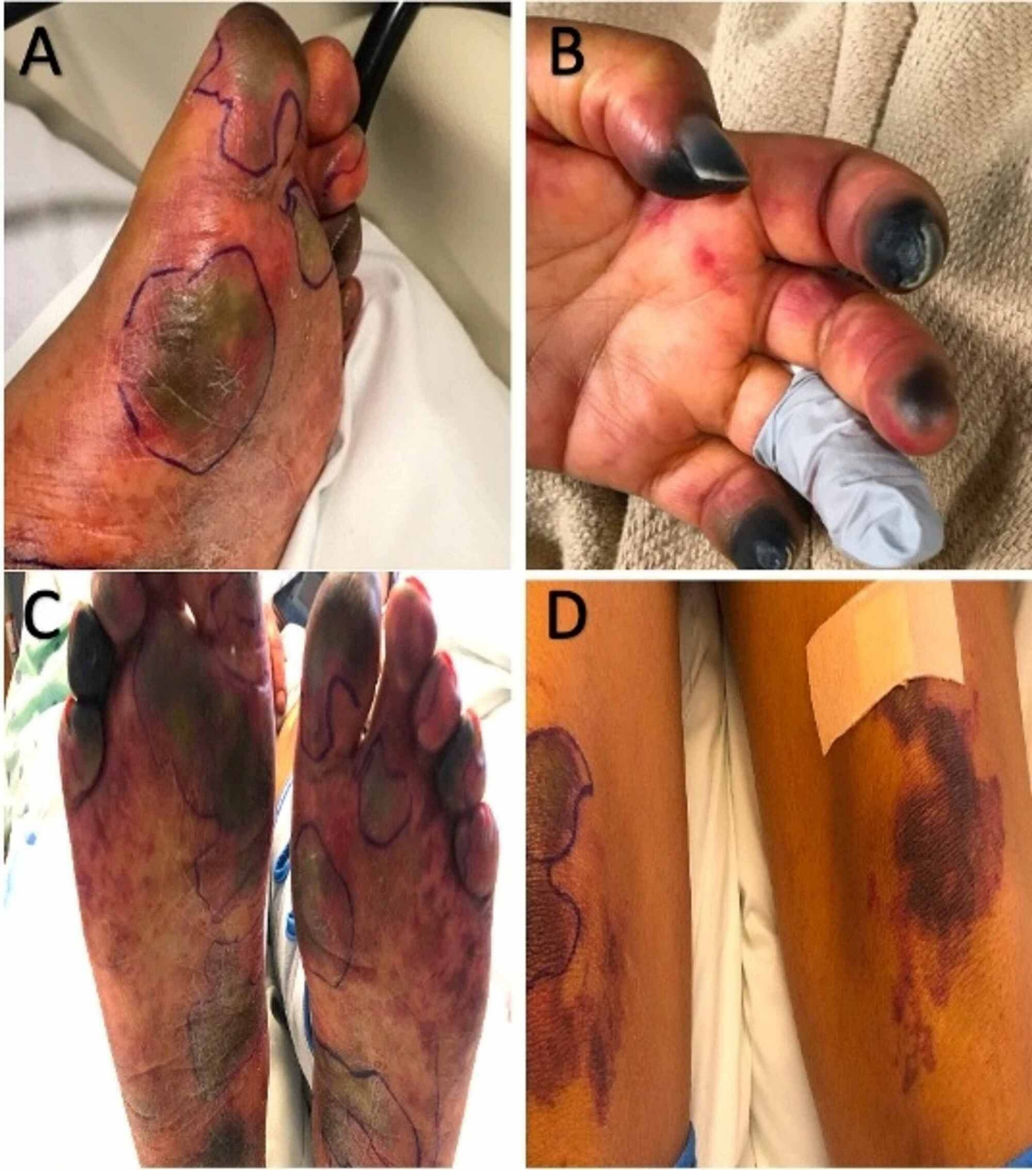
Acral (A-C) lesions with hemorrhagic necrosis and non-acral (D) retiform purpuric lesions consistent with symmetric peripheral gangrene and purpura fulminans.

**Figure 3:**
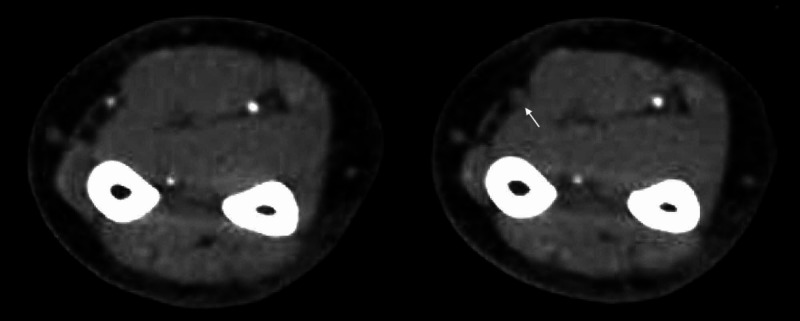
CTA of the left upper extremity demonstrating radial artery occlusion (arrow) and patency of the ulnar artery.

A repeat CT of the abdomen and pelvis demonstrated improvement in the emphysematous pyelonephritis (Figure 1B). Left nephrectomy was therefore not needed. However, several fingers and toes had become gangrenous, and purpura with skin necrosis was noted in bilateral upper and lower extremities (Figure 2). The left radial pulse was not palpable, and no perfusion was observed on arterial doppler ultrasonography. CT angiography confirmed left mid and distal radial artery occlusion (Figure 3) and demonstrated patency of the left ulnar artery, which supplied the palmar arch and digital arteries. Laboratory studies revealed a platelet count of 20,000, fibrinogen of 686 mg/dL, prothrombin time of 24.2 seconds, the international normalized ratio of 2.2, a normal partial thromboplastin time, an elevated haptoglobin, and a peripheral smear with few schistocytes. Vascular surgery was consulted for evaluation, with recommendations for aggressive wound care and to monitor for demarcation of viable tissue. Therapeutic anticoagulation with enoxaparin was initiated following platelet recovery.

**Figure 4:**
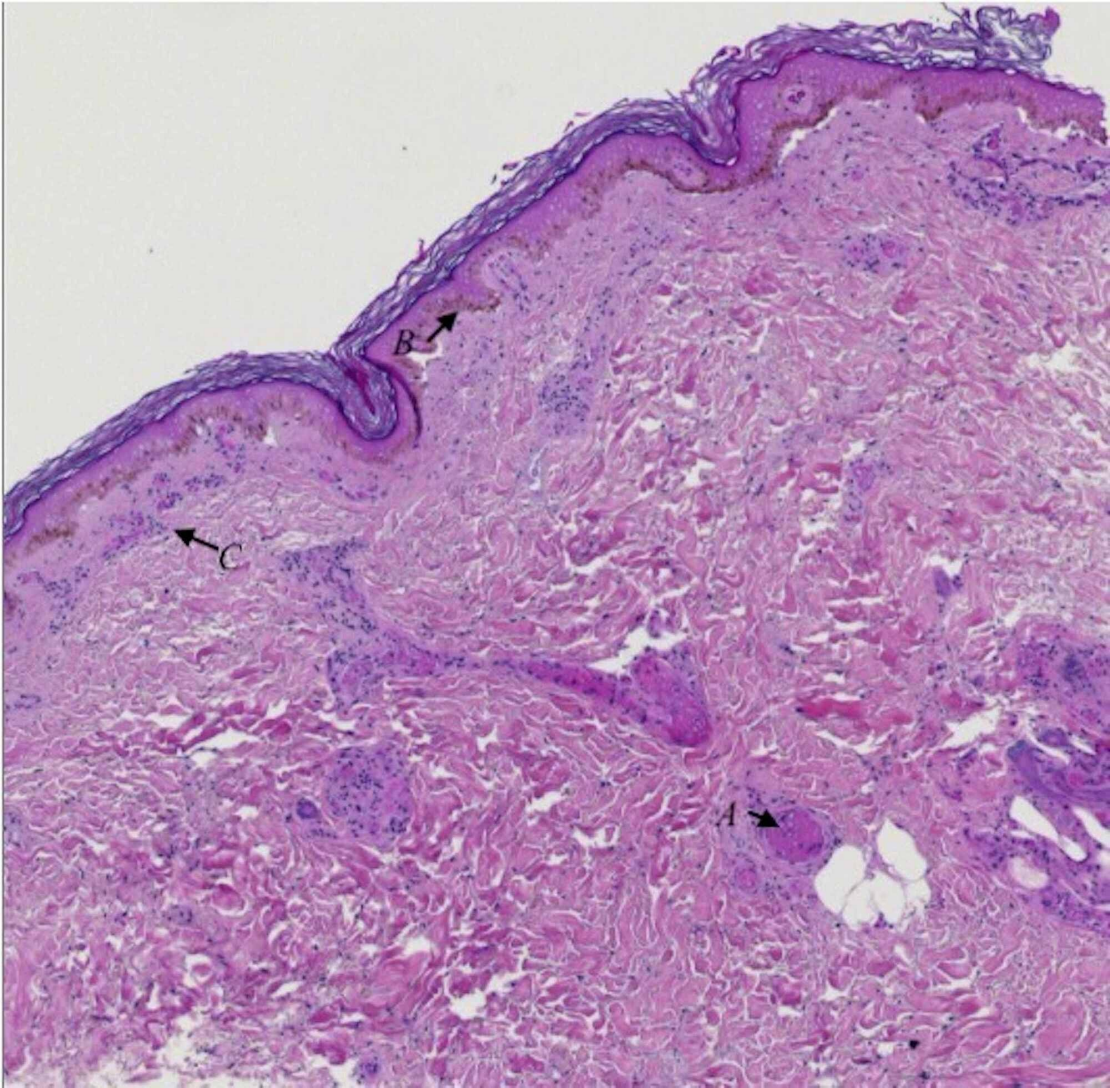
Histologic section shows dermal vessels with fibrin thrombi (arrow A) and both epidermal and adnexal necrosis (arrow B) and mild superficial lymphocytic inflammation (arrow C).

A skin punch biopsy from a lower extremity lesion revealed fibrin thrombi within dermal vessels and epidermal necrosis (Figure 4), findings consistent with DIC with PF. The patient’s platelet counts gradually recovered to within normal range, coinciding with resolving hepatitis, coagulopathy, and acute kidney injury. The decision was made to bridge the patient from enoxaparin to warfarin for long-term anticoagulation prior to discharge. Unfortunately, anticoagulation was not continued in the outpatient setting due to socioeconomic factors. She later developed osteomyelitis in several of the gangrenous digits of the left hand several months later and required partial amputation of these digits and debridement of two fingers of the right hand. Post-operatively, skin examination revealed that purpuric lesions were no longer present and only scattered islands of epidermal necrosis remained on both feet. She has been unable to follow-up with hematology for a comprehensive hypercoagulable evaluation.

## Discussion

The pathogenesis of PF and SPG in patients with severe systemic infections is not well understood, but is thought to involve a decrease in the synthesis of physiologic anticoagulants PC, PS, or antithrombin III (AT3) [5,7], particularly in the context of ischemic hepatitis or “shock liver.” Patients with severe sepsis and septic shock have been shown to have decreased levels of PC and AT3, with the degree of reduction in protein C concentration directly correlated with the severity of sepsis [9]. In addition, superantigens and lipopolysaccharide from Gram-positive and Gram-negative bacteria, respectively, are thought to trigger the release of proinflammatory cytokines that result in shock and thrombosis [10,11]. Overall, PF and SPG are associated with significant morbidity and mortality up to 50%. Individuals often die from overwhelming systemic thrombosis and resultant multi-system organ failure; survivors are often left with scarring of the affected extremities or digits, and amputation is common [6,12]. Prompt recognition of the underlying etiology is of utmost importance to ensure timely management of hemostatic derangements and prevention of organ failure, permanent disability and disfigurement, and death [13].

Thrombocytopenia and purpura in the setting of severe systemic infections, particularly due to *E. coli* as in our patient, should raise concern for several etiologies that warrant the emergent evaluation, including thrombotic thrombocytopenic purpura (TTP), hemolytic uremic syndrome (HUS), and DIC. Our patient had abnormal coagulation studies, and had no evidence of microangiopathy or hemolytic anemia, making TTP or HUS unlikely. Drug-induced thrombocytopenia and immune thrombocytopenic purpura were also unlikely given the presence of thrombocytopenia prior to the administration of any potential causative agent and, given the clinical severity of sepsis, the strong likelihood of an alternative diagnosis; HIV and hepatitis C serologies, as well as antibodies for heparin-induced thrombocytopenia were also negative. While our patient’s laboratory results were not entirely typical of DIC given an elevated fibrinogen level and lack of significant schistocytes (< 1%) on peripheral smear, clinical suspicion remained high and a DIC score was calculated to be 6, with scores >5 having 91% sensitivity and 97% specificity for overt DIC [14].

It is important to rule out other potential etiologies of diffuse thrombosis and purpura, many of which have vastly different management approaches. Our patient’s need for 48 hours of vasoactive support initially led some clinicians to question whether she had simply experienced vasopressor-induced digital ischemia. Notably, our patient presented with shock and ischemic hepatitis, with evidence of microvascular thrombosis several days prior to the administration of vasopressors. Overall, the distribution and time-dependent evolution of the lesions in relation to the onset and duration of shock and ischemic hepatitis makes this consideration unlikely [2,5]. Given the striking features of SPG in our patient, the presence of histologic features of PF on skin punch biopsy of a non-acral lesion further supports our hypothesis of DIC as the primary etiology. PF and SPG have also been associated with lymphoproliferative disorders, myeloproliferative disorders, antiphospholipid syndrome, and pro-inflammatory disorders (i.e., diabetes, peripheral artery disease, Raynaud’s phenomenon, or disease) [2]. However, these conditions are more commonly associated with PF or SPG in the absence of DIC [2]. A comprehensive diagnostic laboratory evaluation and the acuity and progression of the skin lesions made these diagnoses unlikely.

The fundamental strategy to the management of DIC is the aggressive treatment of the inciting cause and optimal supportive care with the use of hemostatic products and anticoagulation based on the clinical context and severity [15-17]. In patients who develop PF or acral ischemia that threaten the integrity of an extremity or digits, early consideration for consultation with a multidisciplinary team of medical, surgical, and wound care specialists is essential to preserve viable tissue and prevent unnecessary amputation [8]. Unfractionated heparin and low-molecular-weight heparin (LMWH) are the agents of choice in the management of DIC where thrombosis predominates [15,18]. Therapeutic levels of anticoagulation should be achieved in these patients and may be monitored either by activated partial thromboplastin time (aPTT) or by anti-factor Xa assay, particularly in patients with baseline aPTT abnormalities [15]. In patients with large-vessel thrombosis, extended anticoagulation may be warranted [4]. While the radial artery is not considered a large-size vessel, we decided on a similar approach for our patient and treated it as such, and planned for long-term outpatient anticoagulation. Taking our patient’s socioeconomic factors into consideration, warfarin was chosen to be bridged with enoxaparin. Bridging with LMWH or intravenous heparin and close observation is essential, for there is a potential risk of exacerbating the thrombosis due to further depletion of physiologic anticoagulants upon initiation of warfarin [4].

An important consideration in the use of heparin or LMWH is that of therapeutic resistance due to acquired PC and antithrombin deficiency. If therapeutic levels of anticoagulation are unable to be achieved despite appropriate administration, there is some data to support the coadministration of FFP to replenish stores of PC and antithrombin consumed by sepsis, particularly in patients with PF and large-vessel thrombosis or central venous catheter thrombosis [4]. This principle has been successfully applied to acquired antithrombin deficiency in adults with septic shock [19] and would provide justification for the use of FFP and LMWH in our patient, who also experienced a hepatic injury as a result of septic shock. Some authors have suggested the potential utility of direct oral anticoagulants in patients with a thrombotic phenotype of DIC, but further investigation is still needed [20]. Medical therapy of DIC is an evolving field of study, with potential therapeutic options including high-dose antithrombin, recombinant soluble thrombomodulin, and therapeutic plasma exchange [12,17].

## Conclusions

The management of DIC manifesting as PF or SPG in patients with severe sepsis and septic shock hinges on early recognition, aggressive treatment of the underlying infection, and optimal supportive care of coagulopathy and organ dysfunction. While patients with DIC are generally at high risk of bleeding, DIC may take on a more thrombotic phenotype and should be considered in the presence of microvascular thrombosis, hemorrhagic necrosis, and gangrene. Patients who develop PF and SPG are best managed with a multidisciplinary team between medical, surgical, and nursing specialists due to the high risk of morbidity and mortality associated with multi-system organ failure and amputation. Anticoagulation with heparin or LMWH is recommended if feasible, with extended courses particularly warranted in patients with large-vessel thrombosis. Additional research is needed for other therapies, including direct oral anticoagulants. This case adds to the growing body of literature implicating *E. coli* infections as a cause of PF and SPG.
